# Exome sequencing identifies germline variants in *DIS3* in familial multiple myeloma

**DOI:** 10.1038/s41375-019-0452-6

**Published:** 2019-04-09

**Authors:** Maroulio Pertesi, Maxime Vallée, Xiaomu Wei, Maria V. Revuelta, Perrine Galia, Delphine Demangel, Javier Oliver, Matthieu Foll, Siwei Chen, Emeline Perrial, Laurent Garderet, Jill Corre, Xavier Leleu, Eileen M. Boyle, Olivier Decaux, Philippe Rodon, Brigitte Kolb, Borhane Slama, Philippe Mineur, Eric Voog, Catherine Le Bris, Jean Fontan, Michel Maigre, Marie Beaumont, Isabelle Azais, Hagay Sobol, Marguerite Vignon, Bruno Royer, Aurore Perrot, Jean-Gabriel Fuzibet, Véronique Dorvaux, Bruno Anglaret, Pascale Cony-Makhoul, Christian Berthou, Florence Desquesnes, Brigitte Pegourie, Serge Leyvraz, Laurent Mosser, Nicole Frenkiel, Karine Augeul-Meunier, Isabelle Leduc, Cécile Leyronnas, Laurent Voillat, Philippe Casassus, Claire Mathiot, Nathalie Cheron, Etienne Paubelle, Philippe Moreau, Yves–Jean Bignon, Bertrand Joly, Pascal Bourquard, Denis Caillot, Hervé Naman, Sophie Rigaudeau, Gérald Marit, Margaret Macro, Isabelle Lambrecht, Manuel Cliquennois, Laure Vincent, Philippe Helias, Hervé Avet-Loiseau, Victor Moreno, Rui Manuel Reis, Judit Varkonyi, Marcin Kruszewski, Annette Juul Vangsted, Artur Jurczyszyn, Jan Maciej Zaucha, Juan Sainz, Malgorzata Krawczyk-Kulis, Marzena Wątek, Matteo Pelosini, Elzbieta Iskierka-Jażdżewska, Norbert Grząśko, Joaquin Martinez-Lopez, Andrés Jerez, Daniele Campa, Gabriele Buda, Fabienne Lesueur, Marek Dudziński, Ramón García-Sanz, Arnon Nagler, Marcin Rymko, Krzysztof Jamroziak, Aleksandra Butrym, Federico Canzian, Ofure Obazee, Björn Nilsson, Robert J. Klein, Steven M. Lipkin, James D. McKay, Charles Dumontet

**Affiliations:** 1 Genetic Cancer Susceptibility, International Agency for Research on Cancer, Lyon, France; 2 Department of Laboratory Medicine, Division of Hematology and Transfusion medicine, Lund University, Lund, Sweden; 3 Biological Statistics and Computational Biology, Cornell University, Ithaca, NY USA; 4 Medicine, Weill Cornell Medical College, New York, NY USA; 5 ProfilExpert, Lyon, France; 6 Hospices Civils de Lyon, Lyon, France; 7 Medical Oncology Service, Hospitales Universitarios Regional y Virgen de la Victoria; Institute of Biomedical Research in Malaga (IBIMA), CIMES, University of Málaga, Málaga, Spain; 8 INSERM 1052, CNRS 5286, CRCL, Lyon, France; 9 University of Lyon, Lyon, France; 10 INSERM, UMR_S 938, Paris, France; 11 AP-HP, Hôpital Saint Antoine, Departement d’hematologie et de therapie cellulaire, Paris, France; 12 Sorbonne Universites, UPMC Univ Paris 06, UMR_S 938, Paris, France; 13 IUC-Oncopole and CRCT INSERM U1037, Toulouse, France; 14 Inserm CIC 1402 & Service d’Hématologie et Thérapie Cellulaire, CHU La Miletrie, Poitiers, France; 15 Hôpital Claude Huriez, CHRU, Lille, France; 16 Service de Medecine Interne, CHU Rennes, Rennes, France; 17 Faculte de Medecine, Universite de Rennes 1, Rennes, France; 18 INSERM UMR U1236, Rennes, France; 19 Unite d’Hematologie et d’Oncologie, Centre Hospitalier, Perigueux, France; 20 Hematologie Clinique, CHU de Reims, Reims, France; 21 Service d’Onco hematologie, CH Avignon, Avignon, France; 22 Hematologie et pathologies de la coagulation, Grand Hôpital de Charleroi, Charleroi, Belgium; 23 Centre Jean Bernard, Institut Inter-regional de Cancerologie, Le Mans, France; 24 Service post urgences, CHU de FORT DE FRANCE, pôle RASSUR, Martinique, France; 25 Hopital Jean Minjoz, CHRU Besançon, Besançon, France; 26 Service d’Hemato-Oncologie, CHU Chartres, Chartres, France; 27 Hematologie clinique et therapie cellulaire, CHU Amiens, Amiens, France; 28 Service de rhumatologie, CHU Poitiers, Poitiers, France; 29 Cancer Genetics Department, Paoli-Calmettes Institute, Aix-Marseille University, Marseille, France; 30 Service d’Immuno-hematologie, Hôpital Saint Louis, Paris, France; 31 Service d’Hematologie, CHU de Nancy, Universite de Lorraine, Vandoeuvre les Nancy, Nancy, France; 32 Internal Medicine Department, Archet Hospital, CHU Nice, Nice, France; 33 Service d’Hematologie, CHR Mercy, Metz, France; 34 Unite d’Hematologie, CH Valence, Valence, France; 35 Service d’Hematologie, Centre Hospitalier Annecy Genevois, Epagny Metz-Tessy, France; 36 Service d’Hematologie, CHU de Brest, Brest, France; 37 Haematology Department, CHU UCL Namur, Yvoir, Belgium; 38 Hematologie clinique, CHU de Grenoble, La Tronche, France; 39 Departement d’oncologie, CHUV, Lausanne, Switzerland; 40 Unite d’oncologie medicale, Pôle medical 2, Hôpital Jacques Puel, Rodez, France; 41 CH Poissy, Saint-Germain-en-Laye, France; 42 Service Hematologie, Institut de Cancerologie Lucien Neuwirth, Saint-Priest-en-Jarez, France; 43 Hematologie, CHG Abbeville, Abbeville, France; 44 Institut Daniel Hollard, Groupe Hospitalier Mutualiste de Grenoble, Grenoble, France; 45 Service hemato/oncologie, CH William Morey, Chalon sur Saône, France; 46 Hematologie clinique, Hôpital Avicenne, Bobigny, France; 47 Intergroupe Francophone du Myelome (IFM), Bobigny, France; 48 Service Hematologie, CH Bligny, Briis-sous-Forges, France; 49 Service Hematologie, CH Lyon Sud, Pierre Benite, France; 50 Service Hematologie, CHU Nantes, Nantes, France; 51 Laboratoire de Biologie Medicale OncoGènAuvergne; Departement d’oncogenetique, UMR INSERM 1240, Centre Jean Perrin, Clermont-Ferrand, France; 52 Service d’hematologie clinique, Pôle medecine de specialite, Centre Hospitalier Sud Francilien (CHSF), Corbeil-Essonnes, France; 53 Hematologie Clinique, CHU Nîmes, Nîmes, France; 54 Hematologie Clinique, CHU Dijon, Dijon, France; 55 Hematologie - Oncologie medicale, Centre Azureen de Cancerologie, Mougins, France; 56 Service d’Hematologie et d’Oncologie, CHU de Versailles, Le Chesnay, France; 57 INSERM U1035, Universite de Bordeaux, Bordeaux, France; 58 Hematologie Clinique, IHBN-CHU CAEN (University Hospital), Caen, France; 59 Rheumatology Department, Maison Blanche Hospital, Reims University Hospitals, Reims, France; 60 Unite d’Hematologie clinique, Groupement des hôpitaux de l’Institut Catholique (GHICL), Universite Catholique de Lille, Lille, France; 61 Departement d’hematologie clinique, CHU de Montpellier, Montpellier, France; 62 Service d’Oncologie medicale, CHU de La Guadeloupe, Pointe-a-Pitre, Guadeloupe; 63 Laboratory for Genomics in Myeloma, Institut Universitaire du Cancer and University Hospital, Centre de Recherche en Cancerologie de Toulouse, Toulouse, France; 64 CIBER Epidemiología y Salud Pública (CIBERESP), Madrid, Spain; 65 Unit of Biomarkers and Susceptibility, Cancer Prevention and Control Program, IDIBELL, Catalan Institute of Oncology; Department of Clinical Sciences, Faculty of Medicine, University of Barcelona, Barcelona, Spain; 66 Life and Health Sciences Research Institute (ICVS), School of Health Sciences, University of Minho, Braga, Portugal; ICVS/3B’s-PT Government Associate Laboratory, Braga/Guimarães, Portugal; 67 Molecular Oncology Research Center, Barretos Cancer Hospital, Barretos, São Paulo Brazil; 68 3rd Department of Internal Medicine, Semmelweis University, Budapest, Hungary; 69 Department of Hematology, University Hospital, Bydgoszcz, Poland; 70 Department of Haematology, Rigshospitalet, Copenhagen University, Copenhagen, Denmark; 71 Jagiellonian University Medical College, Department of Hematology, Cracow, Poland; 72 Gdynia Oncology Center, Gdynia and Department of Oncological Propedeutics, Medical University of Gdańsk, Gdańsk, Poland; 73 Genomic Oncology Area, GENYO. Centre for Genomics and Oncological Research: Pfizer/University of Granada/Andalusian Regional Government, PTS Granada, Granada, Spain; 74 Department of Bone Marrow Transplantation and Hematology-Oncology M. Sklodowska-Curie Memorial Cancer Center and Institute of Oncology Gliwice Branch, Gliwice, Poland; 75 Department of Hematology, Institute of Hematology and Transfusion Medicine, Warsaw, Poland; 76 Holycross Cancer Center of Kielce, Hematology Clinic, Kielce, Poland; 77 Department of Oncology, Transplants and Advanced Technologies, Section of Hematology, Pisa University Hospital, Pisa, Italy; 78 Department of Hematology, Medical University of Lodz, Łódź, Poland; 79 Department of Experimental Hemato-oncology, Medical University of Lubli, Poland; Department of Hematology, St. John’s Cancer Centre, Polish Myeloma Study Group, Lublin, Poland; 80 Hematology Department, Hospital 12 de Octubre, Universidad Complutense; CNIO, Madrid, Spain; 81 Hematology and Medical Oncology Department, Hospital Morales Meseguer, IMIB, Murcia, Spain; 82 Department of Biology, University of Pisa, Pisa, Italy; 83 Inserm U900, Institut Curie, PSL Research University, Mines ParisTech, Paris, France; 84 Teaching Hospital No1, Hematology Dept, Rzeszow, Poland; 85 Hematology Department, University Hospital of Salamanca, IBSAL, Salamanca, Spain; 86 Hematology Division, Chaim Sheba Medical Center, Tel Hashomer, Israel; 87 Department of Hematology, Copernicus Hospital, Torun, Poland; 88 Wroclaw Medical University, Wroclaw, Poland; 89 Genomic Epidemiology Group, German Cancer Research Center (DKFZ), Heidelberg, Germany; 90 Department of Genetics and Genomic Sciences and Icahn Institute for Genomics and Multiscale Biology, Icahn School of Medicine at Mount Sinai, New York, NY USA

**Keywords:** Myeloma, Cancer genetics

## To the Editor

Multiple myeloma (MM) is the third most common hematological malignancy, after Non-Hodgkin Lymphoma and Leukemia. MM is generally preceded by Monoclonal Gammopathy of Undetermined Significance (MGUS) [1], and epidemiological studies have identified older age, male gender, family history, and MGUS as risk factors for developing MM [2].

The somatic mutational landscape of sporadic MM has been increasingly investigated, aiming to identify recurrent genetic events involved in myelomagenesis. Whole exome and whole genome sequencing studies have shown that MM is a genetically heterogeneous disease that evolves through accumulation of both clonal and subclonal driver mutations [3] and identified recurrently somatically mutated genes, including *KRAS*, *NRAS*, *FAM46C*, *TP53*, *DIS3*, *BRAF*, *TRAF3*, *CYLD*, *RB1* and *PRDM1* [3–5].

Despite the fact that family-based studies have provided data consistent with an inherited genetic susceptibility to MM compatible with Mendelian transmission [6], the molecular basis of inherited MM predisposition is only partly understood. Genome-Wide Association (GWAS) studies have identified and validated 23 loci significantly associated with an increased risk of developing MM that explain ~16% of heritability [7] and only a subset of familial cases are thought to have a polygenic background [8]. Recent studies have identified rare germline variants predisposing to MM in *KDM1A* [9], *ARID1A* and *USP45* [10], and the implementation of next-generation sequencing technology will allow the characterization of more such rare variants.

In this study, we sought to explore the involvement of rare germline genetic variants in susceptibility to MM.

Within our discovery cohort of peripheral blood samples (see Supplementary Methods) from 66 individuals from 23 unrelated families analyzed by WES, *DIS3* (NM_014953) was the only gene in which putative loss-of-function variants were observed in at least two families. An additional cohort of 937 individuals (148 MM, 139 MGUS, 642 unaffected relatives and eight individuals with another hematological condition) from 154 unrelated families (including the individuals in the discovery cohort) were screened for germline variants in *DIS3* using targeted sequencing (Supplementary Table S1). In total, we detected *DIS3* germline putative loss-of-function variants in four unrelated families. The *DIS3* genotypes for the identified variants were concordant between WES and targeted sequencing (where available) and independently confirmed by Sanger sequencing on DNA extracted from uncultured whole blood. The variant allele frequencies (VAF) were close to 50%, as expected of a germline variant (Supplementary figure S1).

The *DIS3* gene, located in 13q22.1, encodes for the catalytic subunit of the human exosome complex, and is recurrently somatically mutated in MM patients [4, 5, 11, 12]. The somatic variants are predominantly missense variants localized in the RNB domain mainly abolishing the exoribonucleolytic activity [4, 13], and are often accompanied by LOH or biallelic inactivation due to 13q14 deletion, implying a tumor suppressor role for *DIS3* in MM [5, 12, 13].

**Fig. 1:**
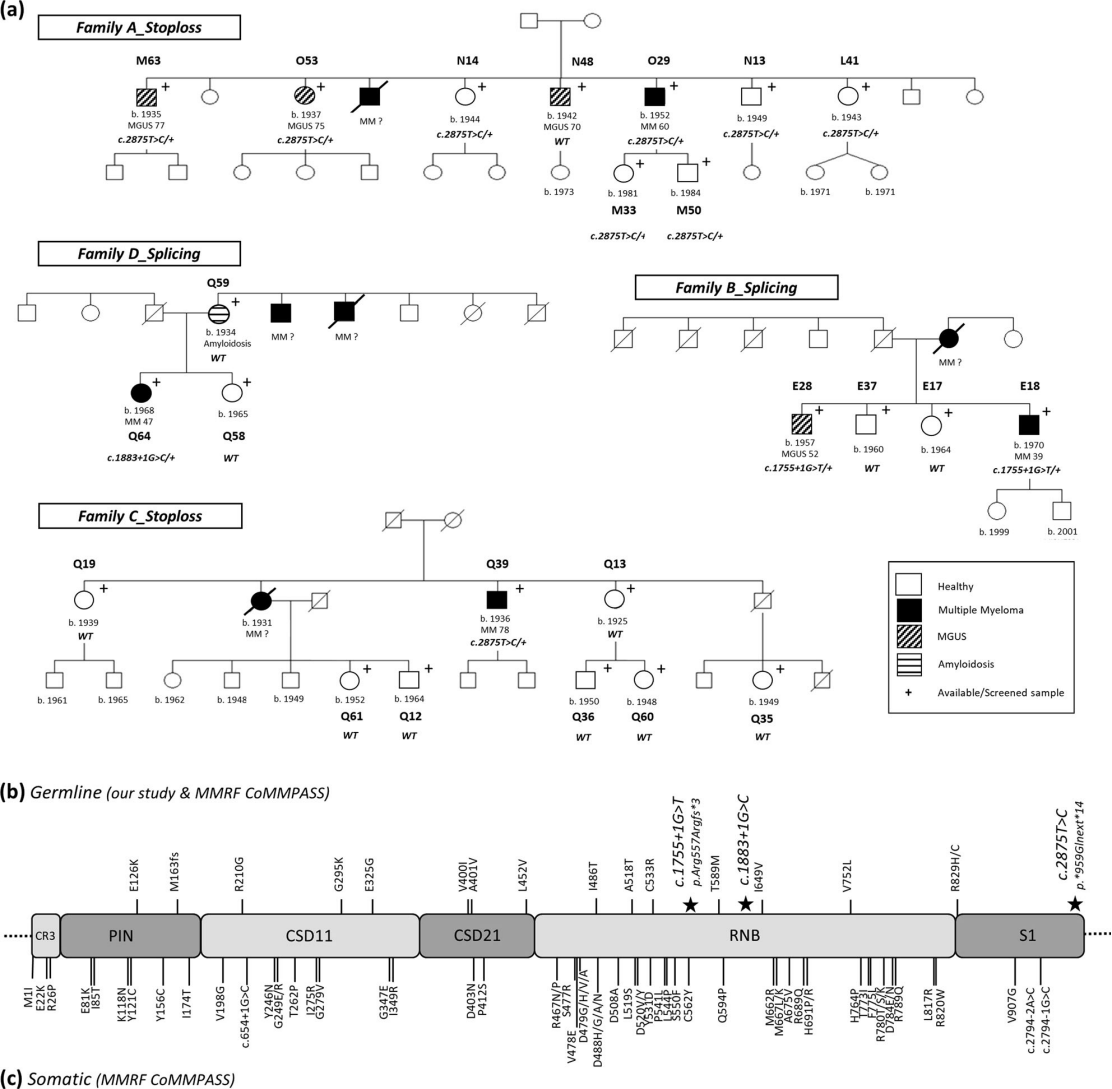
*DIS3* variants in MM cases. **a** Pedigrees from families carrying a germline *DIS3* variant. Available samples for screening are marked with a “+” symbol. Families A and C carry the p.*959Glnext*14 (c.2875T>C) stop-loss variant. Family B carries the c.1755+1G>T splicing variant and family D carries the c.1883+1G>C splicing variant. The genotype of all screened individuals is shown on each pedigree. WT*:* wild type. **b**, **c** Schematic representation of identified germline and somatic variants in the distinct DIS3 protein domains. **b** Germline variants were identified through WES and targeted resequencing in families with reoccurrence of MM/MGUS as well as in a collection of sporadic MM cases (MMRF CoMMpass Study). The *DIS3* variants discussed in the present study are depicted with a star on the upper part of the figure. **c** Somatic *DIS3* variants were identified in sporadic MM cases from the MMRF CoMMpass Study. We observe that in contrast to the clustering of somatic *DIS3* missense variants in the RNB and PIN domains, germline variants are scattered throughout the gene and consist of splicing, stop-loss and missense variants

**Fig. 2:**
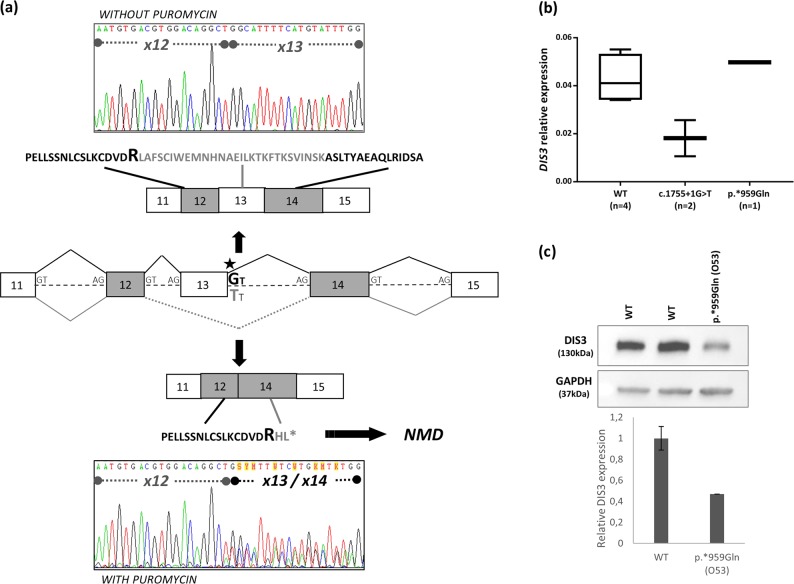
*DIS3* c.1755+1G>T splicing variant results in nonsense-mediated mRNA decay (NMD) and affects mRNA expression, while the c.2875C>T (p.*959Glnext*14) stop-loss variant affects protein levels. **a** LCLs from patients E18 and E28 (not shown) carrying the c.1755+1G>T splicing variant were cultured with and without puromycin. The chromatogram from treated cells (with puromycin) showed a mixture of the wild-type and mutant transcript lacking exon 13, which was not detected in the non-treated cells (without puromycin). Thus, the mutant transcript is degraded by NMD. **b** Box plot representing the relative *DIS3* mRNA expression in c.1775+1G>A (n=2) and p.*959Glnext*14 (*n* = 1) carriers compared to non-carriers (*n* = 4). All reactions were performed in triplicates. **c** Western blot with an anti-DIS3 antibody was performed in LCLs from one p.*959Glnext*14 carrier and two wild-type individuals (anti-GAPDH antibody as internal control). The relative DIS3 expression in the p.*959Glnext*14 carrier was reduced by 50% compared to non-carriers, suggesting that the mutant allele is translated but degraded shortly after

The first *DIS3* variant, observed in 2 affected siblings (1 MGUS and 1 MM case) from family B (Fig. 1a), was located in the splice donor site of exon 13 (c.1755+1G>T; chr13: 73,345,041; GRCh37/hg19, rs769194741) (Supplementary Figure S1a). It is predicted to abolish the splice donor site and cause skipping of exon 13, introducing a premature termination codon (p.Arg557Argfs*3) and result in a truncated DIS3 protein that lacks part of the exonucleolytic active RNB and S1 domains (Fig. 1b, c). The presence of this variant in two siblings, implying Mendelian segregation, is consistent with a germline, rather than somatic, origin. We investigated whether a *DIS3* transcript from the variant allele is generated but is subsequently eliminated by Nonsense Mediated Decay (NMD) by incubating Lymphoblastoid Cell Lines (LCLs) derived from the two c.1755+1G>T allele carriers with and without puromycin, which suppresses NMD. The mRNA transcript corresponding to the variant allele was clearly present in LCLs treated with puromycin in both carriers, whereas not detectable in untreated LCLs (Fig. 2a), consistent with the variant allele being transcribed but subsequently degraded via the NMD pathway. In line with this observation, analysis of *DIS3* mRNA expression by qRT-PCR showed an average 50% reduced expression in the c.1755+1G>T carriers (range 40.7−61.4%) as compared to non-carriers (Fig. 2b). A second splicing variant (c.1883+1G>C; chr13: 73,342,922; GRCh37/hg19) located in the splice donor site of exon 14 within the RNB domain was identified in a MM case from family D (Fig. 1a, b, Supplementary figure S1c). However, the individual’s mother (Q59), affected with amyloidosis, did not carry the variant, implying that MM in the allele carriers’ maternal uncles is unlikely to be explained by this *DIS3* variant. Whether the mRNA transcript encoded by this germline variant undergoes NMD could not be explored due to lack of appropriate material (LCLs, RNA).

A third *DIS3* variant disrupting the wild-type termination codon (stop-loss) (c.2875T>C; p.*959Gln; chr13:73,333,935; GRCh37/hg19, rs141067458) (Fig. 1b, Supplementary Figure S1b) was identified in two unrelated families (A and C, Fig. 1a). This variant is expected to result in a putative read-through variant and a DIS3 protein with an additional 13 amino acids in the C-terminus (p.*959Glnext*14). It was detected in 3 out of 4 affected siblings (2 MGUS (M63, O53) and 1 MM case (O29)), as well as 5 unaffected relatives (N14, N13, L41, M33 M50) from family A. The Mendelian segregation of this variant in this pedigree is also consistent with germline origin. An additional MM case from family C carried the variant, while we were unable to assess the other MM-afflicted family member (Fig. 1a). As expected of a stop-loss variant, NMD was not observed (data not shown), and gene expression analysis showed no effect on *DIS3* mRNA levels (Fig. 2b). However, western blot analysis demonstrated that DIS3 protein levels were markedly lower (~50%) in the p.*959Glnext*14 carrier (O53, family A) compared to non-carriers (Fig. 2b, c).

Next, we sought to determine if rare, putative deleterious variants in *DIS3* were more frequent in an independent series of MM cases compared to unaffected individuals. We performed mutation burden tests between 781 MM cases and 3534 controls from the MMRF CoMMpass Study with WES data available. After testing for systemic bias in this dataset (see Supplementary Methods, Supplementary Figure S2), we undertook a burden test for association between functional *DIS3* variants and MM. *DIS3* putative functional variants (truncating and likely deleterious missense variants, see Supplementary Methods) were more frequent among MM patients (30/781) than controls (72/3534) (OR = 1.92 95%CI:1.25–2.96, *p* = 0.001). Although the p.*959Glnext*14 stop-loss variant was recurrently found in 10/781 MM cases and 15/3534 controls (OR = 3.07 95%CI:1.38 to 6.87, *p* *=* 0.0007), it did not entirely explain the excess of *DIS3* variants among cases as there is evidence for association with other putative functional variants (Supplementary Figure S3a). We additionally genotyped the p.*959Glnext*14 stop-loss variant in an independent series of sporadic MM cases and controls from the IMMEnSE Consortium. While this variant was very rare in this series (8/3020 MM cases relative to 3/1786 controls), there was a consistent but non-significant association between this variant and MM (OR = 3.15 95% CI: 0.74–13.43 *p* *=* 0.122).

To explore the functional consequence of germline *DIS3* variants, we compared MM tumor transcriptomes from patients harboring germline (*n* = 21) and somatic (*n* = 96) *DIS3* putative functional variants to non-carriers (*n* = 655). Differential expression analyses showed an enrichment of pathways associated with global ncRNA processing and translational termination in germline *DIS3* carriers including ncRNA processing, ncRNA metabolic process, translational termination, and RNA metabolism. Among somatic *DIS3* carriers, significantly enriched pathways include interferon alpha/beta signalling, mRNA splicing, mRNA processing and transcription (Supplementary Figure S3b, Supplementary tables S3 and S4a–d). These findings are consistent with the proposed DIS3 role in regulating mRNA processing [14] and more specifically mRNA decay, gene expression and small RNA processing [15]. We also observed that, several long-intergenic non-protein coding RNAs, non-coding and antisense RNAs were significantly enriched among *DIS3* carriers (Supplementary table S5a, b) supporting previous studies that demonstrate an accumulation of transcripts from non-protein coding regions, snoRNA precursors and certain lncRNAs in *DIS3* mutant cells, along a general deregulation of mRNA levels probably due to the sequestration of transcriptional factors from the accumulated nuclear RNAs [16].

To our knowledge, this is the first observation of germline *DIS3* likely deleterious variants in familial MM and our results suggest that the involvement of *DIS3* in MM etiology may extend beyond somatic alterations to germline susceptibility. We reported rare germline *DIS3* variants in ~2.6% of our cohort of families with multiple cases of MM and MGUS (4/154). The germline variants described here are predicted to have loss-of-function impact on DIS3. Consistent with this, the 1755+1G>T (rs769194741) splicing variant induces NMD and results in reduced *DIS3* mRNA expression, supporting the proposal that *DIS3* is acting as a tumor suppressor gene in MM [13]. Moreover, the c.2875T>C (rs141067458) stop-loss variant (p.*959Glnext*14) results in reduced DIS3 protein expression suggesting that the mutant allele is translated but degraded shortly after. Notably, in contrast to the clustering of somatic *DIS3* mutations in the PIN and RNB domains, germline variants identified both in familial and sporadic MM cases are scattered throughout the gene (Fig. 1b, c). Despite the fact that these variants do not segregate perfectly with MM in the identified families and the rarity of *DIS3* germline likely deleterious variants limits our statistical power, the subsequent mutation burden and transcriptome analyses provided supportive data towards *DIS3* acting as an “intermediate-risk” MM susceptibility gene.

## Supplementary information

18-LEU-1250_RevisedManuscript_SupplementaryData
