# Supplementary material for: Exome sequencing identifies germline variants in DIS3 in familial multiple myeloma
Source: Leukemia. 2019 Apr 9;33(9):2324–30. doi: 10.1038/s41375-019-0452-6 (PMC6756025; doi:10.1038/s41375-019-0452-6)
Supplement: Supplementary file 1 — 18-LEU-1250_RevisedManuscript_SupplementaryData [file 41375_2019_452_MOESM1_ESM.pdf]

SUPPLEMENTARY APPENDIX

Supplementary Methods

Supplementary Tables

Table S1. Family characteristics.

Table S2. Clinical characteristics of *DIS3* mutation carriers among familial and sporadic MM cases.

Table S3. Selected significant GO and REACTOME categories (q-value < 0.05, NES > 0.5) enriched in carriers of *DIS3* likely deleterious variants (germline, somatic). *originalGeneSetSize*: GO/REACTOME term size from MSigDB, *actualGeneSetSize*: size of the genes from the GO/REACTOME term found in the dataset, *NES*: Normalized Enrichment Score.

Table S4. Mann-Whitney-Wilcoxon Gene Set Test (MWW-GST) analysis of GO and REACTOME categories. **(a,b)** Significant GO and REACTOME categories (q-value < 0.05 and NES > 0.5) enriched in carriers of germline likely deleterious *DIS3* variants (*n*=21) from the Mann-Whitney-Wilcoxon-GST enrichment analysis. **(c,d)** Significant GO and REACTOME categories (q-value < 0.05 and NES > 0.5) enriched in carriers of somatic likely deleterious *DIS3* variants (*n*=96) from the Mann-Whitney-Wilcoxon-GST enrichment analysis. *originalGeneSetSize* is the GO term size from MSigDB; *actualGeneSetSize* is the size of the genes from the GO term found in the dataset; *NES* is the Normalized Enrichment Score.

Table S5. Ranked gene list from **(a)** 21 carriers of germline likely deleterious *DIS3* variants versus 655 non-carriers and **(b)** 96 carriers of somatic likely deleterious *DIS3* variants versus 655 non-carriers. ee-MWW-score is the score associated to each gene resulting from the ee-MWW procedure.

Table S6. Characteristics of MM cases and controls from the IMMEnSE consortium.

Supplementary Figures

Figure S1. IGV plots and Sanger sequencing chromatograms showing the identified *DIS3* variants: **(A)** c.1755+1G>T, **(B)** c.2875T>C (p.*959Glnext*14) and **(C)** c.1883+1G>C. Median Variant Allele Frequency (VAF), where available, is shown for each variant.

Figure S2. Quantile-quantile plots of gene-based burden tests for **(a)** rare neutral synonymous variants and **(b)** rare predicted deleterious variants across genes assessed by

WES. Lambda statistics are shown on the plots. Logistic regression, with adjustment for ancestry inferred by genotype, was used to test for association between burden with individuals being defined as exposed if they carry one (or more) variants (synonymous or deleterious).

Figure S3. Mutation burden test and transcriptome analysis in carriers of *DIS3* variants. **(a)** Burden test association results for MM cases and controls carrying *DIS3* germline likely deleterious variants. OR is adjusted for ancestry inferred by genotype. **(b)** ee-MWW enrichment plots for selected significantly enriched GO and REACTOME pathways among carriers of germline likely deleterious *DIS3* variants (GO pathway Translational Termination, GO pathway ncRNA Processing, GO pathway ncRNA metabolic process and REACTOME pathway Metabolism of RNA). NES and q-values are indicated. Marked in red are genes involved in the respective pathways relative to other genes (in grey).

Supplementary Methods

Study population (MM/MGUS families)

Families with at least two cases of dysglobulinemia (defined as MGUS or MM) were clinically identified through the Intergroupe Francophone du Myélome (IFM), a large cooperative group with >100 centres in France, Belgium and Switzerland. The study was approved by the Hospices Civils de Lyon institutional review board and participants signed an informed consent form.

The recruitment of eligible families started in October 2007, and as of December 2015, 154 non-related families with MM and/or MGUS were enrolled in the study and a total of 937 peripheral blood samples obtained. Diagnosis of MM and MGUS was based on Serum Protein Electrophoresis (SPE) and Immunoelectrophoresis. The majority of recruited families were of European descent, while five families (5/154, 3.2%) originated from the Caribbean island of Martinique. Lymphoblastoid cell lines were established for a subset of 865 samples as previously described[1]. The median number of cases of dysglobulinemia within these families was 2.0 (range 2-6). From the 240 MM and 153 MGUS cases identified in the pedigrees, only 148 and 139 were available for blood sampling, respectively. Additionally, samples from six cases of Waldenström Macroglobulinemia (WM), one case of light chain amyloidosis, one case of lymphoma, as well as 642 unaffected relatives were obtained. Two MM cases were also affected with amyloidosis, and two MGUS patients were affected with lymphoma and Chronic Lymphocytic Leukemia (CLL), respectively. The mean age at recruitment was 66.0 years (range 39-91) for MM cases and 65.4 years (range 35-90) for MGUS patients. The gender ratio (male/female) for MM and MGUS were 1.08 and 0.86, respectively. Family characteristics are shown in **Supplementary table S1**.

Whole exome sequencing was performed on a subgroup of 25 MM, 40 MGUS and 1 WM cases from 23 non-related families with more than two cases of MM and/or MGUS and available material from most of the cases. Additionally, using a targeted resequencing approach we screened 937 samples available from our selection of 154 unrelated families, including 148 MM, 139 MGUS, as well as 642 unaffected family members, and eight individuals with another hematological condition.

Whole exome sequencing (WES) and targeted resequencing applications, validation of sequencing variants

DNA from whole blood (n=72) or lymphoblastoid cell lines (LCLs, after 1 to 9 *in vitro* passages n=865)) was extracted using the QIAmp DNA Mini Kit (Qiagen) and quantified with the Qubit dsDNA BR Assay kit (Thermofischer Scientific).

Germline DNA (1 µg) extracted from whole blood (n=1) and from LCLs after 8 (n=1) and 9 (n=64) passages *in vitro* was used for whole-exome sequence capture using the TruSeq™ Exome Enrichment Kit (Illumina). Pooled libraries were paired-end sequenced (2x100 reads) using the TruSeq™ SBS Kit version 3 on an Illumina HiSeq 2500 (Illumina). Image analysis and base calling was performed with the Illumina RTA software version 1.13.48.

Targeted resequencing of the *DIS3* coding region was performed using a GeneRead DNAseq Custom Panel (QIAGEN), according to the manufacturer's recommendations. The amplicon library was prepared using the NEBNext® Fast DNA Library Prep Set for Ion Torrent™ (New England Biolabs) with custom Ion Xpress Barcode adapters (1-384). Barcoded libraries were quantified (Qubit®) and pooled equimolarly, gel extracted and quantified on a Bioanalyzer High Sensitivity DNA Kit (Agilent technologies). Template preparation by emulsion PCR was performed using the Ion PI Template OT2 200 kit v3 and Ion One Touch 2 system (Thermofischer Scientific) and the positive Ion Sphere™ particles (ISP) were isolated on the Ion OneTouch™ ES module. Templated ISPs were sequenced on an Ion PI™ Chip Kit v2 using the Ion PI™ Sequencing 200 Kit v3 on the Ion Proton™ System (ThermoFisher scientific). The targeted capture covered 98% of *DIS3* coding sequence, with a median coverage of 151X (117X-189X). *DIS3* variants identified by WES and targeted sequencing were technically validated using Sanger sequencing on an independent PCR amplification on DNA extracted from uncultured whole blood.

Data analysis and variant filtering

Alignment of WES raw data on the reference human genome (hg19) was performed using Burrows-Wheeler Aligner (BWA)[2] and sorted with Picard SortSam (http://picard.sourceforge.net),

while duplicate reads were flagged by Picard MarkDuplicates. The Genome Analysis Toolkit (GATK)[3–5] was used for local realignment and base score recalibration of the output files before variant calling. Single nucleotide variants (SNV) and indels were called using GATK UnifiedGenotyper. Variants were subsequently annotated with AnnoVar (version 2013, Aug 23)[6] to describe the frequency of variants in different populations, their position related to genes, if they are likely to result in a coding sequence change and its predicted severity (including assessment of various *in silico* scores for missense variants) and other related attributes. All variants that did not fulfil our quality control criteria (call quality, coding variant, strand bias, segmental duplications, presence of different starting point of reads for the variant allele), along with those reported in public databases (NHLBI GO Exome Sequencing Project (ESP6500)[7], 1000 genomes project[8]), and in a panel of 500 germline TCGA[9] samples at a frequency above 1%, were filtered out. Finally, we only further considered variants that were shared by the majority of affected members within a given family.

Alignment of targeted resequencing reads to the reference genome (hg19) and base calling was performed using Torrent Suite software v3.6.2 (Thermofischer Scientific). Identification of sequence variants was facilitated using FreeBayes[10] and annotated as described above.

Inhibition of Nonsense-Mediated mRNA Decay by puromycin treatment

Lymphoblastoid cell lines (LCLs) from individuals N48 and O53 (family A) and E18 and E28 (family B), heterozygous for the variants of interest, were split evenly in 2 subcultures and one of them was treated with puromycin at a concentration of 100ug/ml (Sigma). Puromycin is a protein synthesis inhibitor that enriches for transcripts harboring a premature stop codon (PTC) through inhibiting their degradation by nonsense-mediated mRNA decay (NMD). After 6 hours of incubation, cells from both treated and untreated subcultures were harvested and total RNA was isolated using the RNeasy Kit (Qiagen). cDNA synthesis was performed using the Superscript III First-strand Synthesis System for RT-PCR (Invitrogen) using oligo-dT primers. *DIS3* exons 10-16 were amplified using specific primers located within exonic regions (ex10F: 5’-AGAAGACCTGAGGCATCTGTG-3’, ex16R: 5’-GAGCAGGATGTTTTCGAAGC-3’), and PCR products were analysed by direct Sanger sequencing.

Quantitative real-time PCR

Total RNA was purified from LCLs for two carriers of the c.1755+1G>T splicing variant (E18, E28), one carrier of the p.*959Glnext*14 stop-loss variant (O53), and four non-carriers (E17, E37, N48 and Q13) from families A, B and D (**Figure 1a**) using the RNeasy mini kit (Qiagen). cDNA was generated using the Omniscript cDNA synthesis kit (Qiagen) and random hexamers. Quantitative PCR (qPCR) was carried out using TaqMan probe-based chemistry on a StepOnePlus Real-Time PCR system (Thermofischer Scientific); Hs00209014_m1 for *DIS3* and Hs02758991_g1 for *GAPDH* as endogenous control. Amplification reactions were performed in triplicates, and data were collected and analysed using the StepOne™Real-Time PCR Software v2.0. The relative quantification in gene expression was determined using the ΔΔCt method.

Western blot

Cells from a carrier of the p.*959Glnext*14 stop-loss variant (O53) and 2 non-carriers were lysed with RIPA buffer supplemented with protease inhibitor cocktail. Western blot was performed using 30 μg of protein and primary antibodies against DIS3 (PA558723, Thermofischer scientific) at 1:150 dilution and GAPDH (6C5, Santa Cruz Biotechnology) at 1:3000 dilution. HRP-conjugated anti-rabbit and anti-mouse secondary antibodies (GE Healthcare) were respectively used at 1:3000 dilution. Immunoreactive proteins were visualized using the Clarity ECL kit on a ChemiDoc XRS+ system (Bio-Rad). Calculations were made using BioRad's Image Lab software v 5.2.

Replication study and Mutation Burden Analysis

Germline WES data from sporadic MM cases (study accession phs000348 and phs000748) (MMRF CoMMpass Study) and controls (study accession phs000209, phs000276, phs000296,

phs000298, phs000424, phs000654, phs000687, phs000806 and phs000878) were obtained from dbGaP. After removing related individuals and restricting to individuals of European origin by principal components analysis (PCA) of common variants, 781 cases and 3534 controls were further analysed. Exomes were jointly called as previously described[11,12]. We focused on rare germline variants (MAF<0.01) in both cases and controls.

For mutation burden testing, loss-of-function (frameshift insertion or deletion, stop-gain, stop-loss, splice-site change) and missense variants with Phred CADD (Combined Annotation Dependent Depletion) score ≥20 were used. For neutral synonymous variant testing, synonymous variants with MAF ≤0.01 were used. The sum of predicted deleterious variants or neutral synonymous coding variant carriers for each gene in MM cases and controls were compared using logistic regression. To eliminate potential spurious associations due to population stratification, we used the first three PCs derived from the PCA testing as covariates in the logistic regression test. Logistic regression and lambda statistics were implemented using R package stats version 3.3.2. We first tested for systemic bias in this dataset by testing for burden association tests based on synonymous variants in genes across the genome. These burden test statistics, not expected to be associated with MM, were consistent with that expected by chance ((genomic inflation (λ) = 0.998)) (**Supplementary Figure S2**).

SNP genotyping

The rare *DIS3* stop-loss variant p.*959Gln (rs141067458) was genotyped in 3020 sporadic MM cases and 1786 controls from seven European countries (Denmark, France, Hungary, Italy, Poland, Portugal, and Spain) and Israel from the International Multiple Myeloma rESEarch (IMMEnSE) Consortium. The characteristics of IMMEnSE cases and controls are shown in **Supplementary table S6**. Genotyping was performed using the KASPar® PCR SNP genotyping system (KBioscience) with a call rate of >84%. Eight percent of the samples were duplicated for quality control and the genotypes showed >99% concordance. Genotype distributions in controls respected Hardy-Weinberg equilibrium. To facilitate the genotyping of this rare variant, established heterozygote variant carriers were included in each genotyping plate, and heterozygote samples were resolved relative to the position of the known samples. Ambiguous samples were

classified as "unknown" and excluded. Genotypes were assigned with the operator blinded to case control status in order to minimize the risk of bias due to technical reasons. Association with MM risk was assessed using unconditional logistic regression adjusting for country of origin (study), age and sex. *P*-values were two sided.

Transcriptome analysis

WES data on both germline and tumor material were used to determine carriers of *DIS3* variants prior to transcriptome analysis. Transcriptome analysis of MMRF CoMMpass MM tumor transcriptomes from carriers of germline likely deleterious *DIS3* variants (n=21), somatic *DIS3* carriers (n=96) and non-carriers (n=655) was performed using the ee-MWW method as previously described[13] and obtained an enrichment score (ES) for each gene. Pathway enrichment analysis was performed using GO Biological Process and REACTOME gene sets ontologies. Pathways with q-value < 0.05 and normalized enrichment score (NES) > 0.5 were considered significant.

Supplementary Tables

Table S1. Family characteristics

Nb of families		***154***
	only MM (*N≥2)*	63 [a]
	only MGUS (*N≥2)*	19 [b]
	MM (*N≥1*) and MGUS (*N≥1*)	72 [c]
MM cases		***240***
	**Available**	148
	- Male	77
	- Female	71
	**Mean age at inclusion**	
	- Male	64.8 (39-91)
	- Female	67.3 (45-87)
	**Heavy chain**	
	- IgA	22
	- IgG	91
	- n/a	35
	**Light chain**	
	- kappa	93
	- lambda	35
	- kappa/lambda	2
	- n/a	18
MGUS cases		***153***
	**Available**	139
	- Male	65
	- Female	74
	**Mean age at inclusion**	
	- Male	65.1 (38-90)
	- Female	65.6 (35-88)
	**Heavy chain**	
	- IgA	16
	- IgG	85
	- IgA/IgG	3
	- IgM	24
	- IgM/IgG	1
	- n/a	10
	**Light chain**	
	- kappa	74
	- lambda	50
	- kappa/lambda	4
	- n/a	11

[a] *One case of amyloidosis was detected within MM only families.*
[b] *Six cases of WM were identified within MGUS only families.*
[c] *One lymphoma case was identified in families with both MM and MGUS cases.*

Table S2. Clinical characteristics of *DIS3* mutation carriers among familial and sporadic MM cases.

Sample	*DIS3 variant*	*Diagnosis*	*Age of onset*	*Family history (Cancers)*	*Heavy chain*	*Light chain*
Familial MM cases (n=7)						
O29_family A	c.2875T>C; p.*959Glnext*14	MM	60[a]	Yes *(MM, MGUS)*	IgG	kappa
M63_family A	c.2875T>C; p.*959Glnext*14	MGUS	77[a]	Yes *(MM, MGUS)*	IgG	kappa, lambda
O53_family A	c.2875T>C; p.*959Glnext*14	MGUS	75[a]	Yes *(MM, MGUS)*	IgG	kappa
E18_family B	c.1755+1G>T	MM	39[a]	Yes *(MM, MGUS)*	IgG	kappa
E28_family B	c.1755+1G>T	MGUS	52[a]	Yes *(MM, MGUS)*	IgM	kappa
Q64_family C	c.1883+1G>C	MM	47[a]	Yes *(MM, amyloidosis)*	n/a	n/a
Q39_family D	c.2875T>C; p.*959Glnext*14	MM	78[a]	Yes *(MM)*	n/a	n/a
MMRF CoMMpass germline DIS3 carriers (n=30)						
MMRC0318	c.376G>A; p.Glu126Lys	MM	n/a	n/a	n/a	n/a
MMRC0499	c.487dupA; p.Met163fs	MM	n/a	n/a	n/a	n/a
MMRC0588	c.487dupA; p.Met163fs	MM	n/a	n/a	n/a	n/a
MMRF_2401	c.628C>G; p.Arg210Gly	MM	64	Unknown	n/a	n/a
MMRF_1659	c.974A>G; p.Glu325Gly	MM	n/a	n/a	n/a	n/a
MMRC0461	c.1198G>A; p.Val400Ile	MM	n/a	n/a	n/a	n/a
MMRC0337	c.1202C>T; p.Ala401Val	MM	n/a	n/a	n/a	n/a
MMRF_1167	c.1202C>T; p.Ala401Val	MM	74	Unknown	n/a	n/a
MMRF_1677	c.1202C>T; p.Ala401Val	MM	50	Unknown	n/a	n/a
MMRF_1808	c.1202C>T; p.Ala401Val	MM	56	Unknown	n/a	n/a
MMRC0543	c.1354C>G; p.Leu452Val	MM	n/a	n/a	n/a	n/a
MMRF_2611	c.1457T>C; p.Ile486Thr	MM	66	Yes *(Lung)*	n/a	n/a
MMRF_1900	c.1552G>A; p.Ala518Thr	MM	77	Yes *(Lung, Prostate, Gynecological Ca)*	n/a	n/a

MMRF_2326	c.1597T>C; p.Cys533Arg	MM	72	No	n/a	n/a
MMRF_1865	c.1766C>T; p.Thr589Met	MM	56	No	n/a	n/a
MMRF_1547	c.1945A>G; p.Ile649Val	MM	65	Yes *(Colorectal)*	n/a	n/a
MMRF_1816	c.2254G>C; p.Val752Leu	MM	66	No	n/a	n/a
MMRF_1309	c.2485C>T; p.Arg829Cys	MM	n/a	n/a	n/a	n/a
MMRF_1768	c.2485C>T; p.Arg829Cys	MM	64	Yes *(Bladder, Breast, Bone, Thyroid)*	n/a	n/a
MMRF_2634	c.2486G>A; p.Arg829His	MM	n/a	n/a	n/a	n/a
MMRC0627	c.2875T>C; p.*959Glnext*14	MM	n/a	n/a	n/a	n/a
MMRC0635	c.2875T>C; p.*959Glnext*14	MM	n/a	n/a	n/a	n/a
MMRC0578	c.2875T>C; p.*959Glnext*14	MM	n/a	n/a	n/a	n/a
MMRF_2074	c.2875T>C; p.*959Glnext*14	MM	61	No	n/a	n/a
MMRF_2213	c.2875T>C; p.*959Glnext*14	MM	63	Unknown	n/a	n/a
MMRF_2253	c.2875T>C; p.*959Glnext*14	MM	61	Yes *(Melanoma, Prostate, Breast)*	n/a	n/a
MMRF_2273	c.2875T>C; p.*959Glnext*14	MM	69	Unknown	n/a	n/a
MMRF_2557	c.2875T>C; p.*959Glnext*14	MM	83	No	n/a	n/a
MMRF_1061	c.2875T>C; p.*959Glnext*14	MM	70	No	n/a	n/a
MMRF_1285	c.2875T>C; p.*959Glnext*14	MM	75	Yes *(Lung)*	n/a	n/a

[a] *For the familial cases age of inclusion in the study is given instead of age of onset*

Table S3. Selected significant GO and REACTOME categories (q-value < 0.05, NES > 0.5) enriched in carriers of *DIS3* likely deleterious variants (germline, somatic). *originalGeneSetSize*: GO/REACTOME term size from MSigDB, *actualGeneSetSize*: size of the genes from the GO/REACTOME term found in the dataset, *NES*: Normalized Enrichment Score

**Germline *DIS3* mutation carriers (n=21)**					
**Category**	**originalGeneSetSize**	**actualGeneSetSize**	**NES**	**q-value**	**Genes**
GO_CELL_CELL_ADHESION_VIA_PLASMA_MEMBRANE_ ADHESION_MOLECULES	204	191	0,626	3,00E-06	Protocadherin alpha gene cluster *(PCDHA1-14)*
GO_OXIDATIVE_PHOSPHORYLATION	84	81	0,677	6,40E-05	*NDUFV1, NDUFB5, NDUFA3, NDUFS2, NDUFS7*
GO_TRANSLATIONAL_TERMINATION	92	89	0,664	1,49E-04	*MRPL34, MRPL17, MRPS25, MRPL49, MRPS30, MRPS23, MRPS12*
GO_NCRNA_PROCESSING	386	360	0,572	4,95E-03	*RRS1, RPUSD2, TSEN34, TRMT12, CPSF4, IMP3, GRSF1*
GO_NCRNA_METABOLIC_PROCESS	533	497	0,560	7,70E-03	*RRS1, RPUSD2, POLR2G, POLR2J, TSEN34, TRMT12, CPSF4, IMP3, GRSF1*
REACTOME_3_UTR_MEDIATED_TRANSLATIONAL_ REGULATION	176	131	0,612	2,35E-03	*EIF3K, EIF3G, EIF3D, EIF3H, RPS26, RPL15, RPL17, RPL14*
REACTOME_NONSENSE_MEDIATED_DECAY_ ENHANCED_BY_THE_EXON_JUNCTION_COMPLEX	176	130	0,603	1,31E-02	*CASC3, RPS26, UPF3A, RPSA, RPL15, RPL14*
REACTOME_METABOLISM_OF_MRNA	284	230	0,571	4,87E-02	*PSMD8, PSMC4, EXOSC7, PSMB8, NCBP2, PSMD4*

**Somatic *DIS3* mutation carriers (n=96)**					
**Category**	**originalGeneSetSize**	**actualGeneSetSize**	**NES**	**q-value**	**Genes**
REACTOME_INTERFERON_ALPHA_BETA_SIGNALING	64	63	0,662	2,00E-03	*IFI6, IFIT3, OASL, IFIT1, IFIT2*
REACTOME_PROCESSING_OF_CAPPED_INTRON_ CONTAINING_PRE_MRNA	140	132	0,612	2,26E-03	*HNRNPR, PCF11, PCBP1, HNRNPU, HNRNPC*
REACTOME_TRANSCRIPTION	210	168	0,597	3,33E-03	*PCF11, HIST2H2AC, HIST4H4, HIST2H2BE, POLR3GL*
REACTOME_MRNA_SPLICING	111	104	0,620	5,53E-03	*HNRNPR, HNRNPU, HIST2H2AC, HIST4H4, HIST1H4J, POLR3GL*
REACTOME_MRNA_PROCESSING	161	152	0,594	1,37E-02	*HNRNPR, HNRNPU, HNRNPC, PCF11, PCBP1*

Table S4. Mann-Whitney-Wilcoxon Gene Set Test (MWW-GST) analysis of GO and REACTOME categories. **(a,b)** Significant GO and REACTOME categories (q-value < 0.05 and NES > 0.5) enriched in carriers of germline likely deleterious *DIS3* variants (*n*=21) from the Mann-Whitney-Wilcoxon-GST enrichment analysis. **(c,d)** Significant GO and REACTOME categories (q-value < 0.05 and NES > 0.5) enriched in carriers of somatic likely deleterious *DIS3* variants (*n*=96) from the Mann-Whitney-Wilcoxon-GST enrichment analysis. *originalGeneSetSize* is the GO term size from MSigDB; *actualGeneSetSize* is the size of the genes from the GO term found in the dataset; *NES* is the Normalized Enrichment Score.

Separate file as dropbox link

https://www.dropbox.com/s/zoxlgg0mhqkvd2q/SupplementaryTable%20S4.xlsx?dl=0

Table S5. Ranked gene list from **(a)** 21 carriers of germline likely deleterious *DIS3* variants versus 655 non-carriers and **(b)** 96 carriers of somatic likely deleterious *DIS3* variants versus 655 non-carriers. ee-MWW-score is the score associated to each gene resulting from the ee-MWW procedure.**Table S6.** Characteristics of MM cases and controls from the IMMEnSE consortium.

Separate file as dropbox link

https://www.dropbox.com/s/7fjriw4k4rjhrrp/Supplementary%20Table%20S5.xlsx?dl=0

Table S6. Characteristics of MM cases and controls from the IMMEnSE consortium.

	Cases	%	Controls	%	Total	%
Age						
Median	62		51			
25-75 percentile	55-68		40-63			
Gender						
Males	1414	52.1%	925	51.8%	2339	52.3%
Females	1302	47.9%	861	48.2%	2163	47.7%
Total	**2716**	**100.0%**	**1786**	**100.0%**	**4502**	**100.0%**
Country						
Italy	299	9.9%	231	12.9%	530	11.0%
Poland	1231	40.8%	342	19.1%	1573	32.7%
Spain	282	9.3%	163	9.1%	445	9.3%
France	501	16.6%	176	9.9%	677	14.1%
Portugal	145	4.8%	192	10.8%	337	7.0%
Hungary	153	5.1%	100	5.6%	253	5.3%
Denmark	300	9.9%	486	27.2%	786	16.4%
Israel	109	3.6%	96	5.4%	205	4.3%
Total	**3020**	**100.0%**	**1786**	**100.0%**	**4806**	**100.0%**

Supplementary Figures

Figure S1. IGV plots and Sanger sequencing chromatograms showing the identified *DIS3* variants: **(A)** c.1755+1G>T, **(B)** c.2875T>C (p.*959Glnext*14) and **(C)** c.1883+1G>C. Median Variant Allele Frequency (VAF), where available, is shown for each variant.

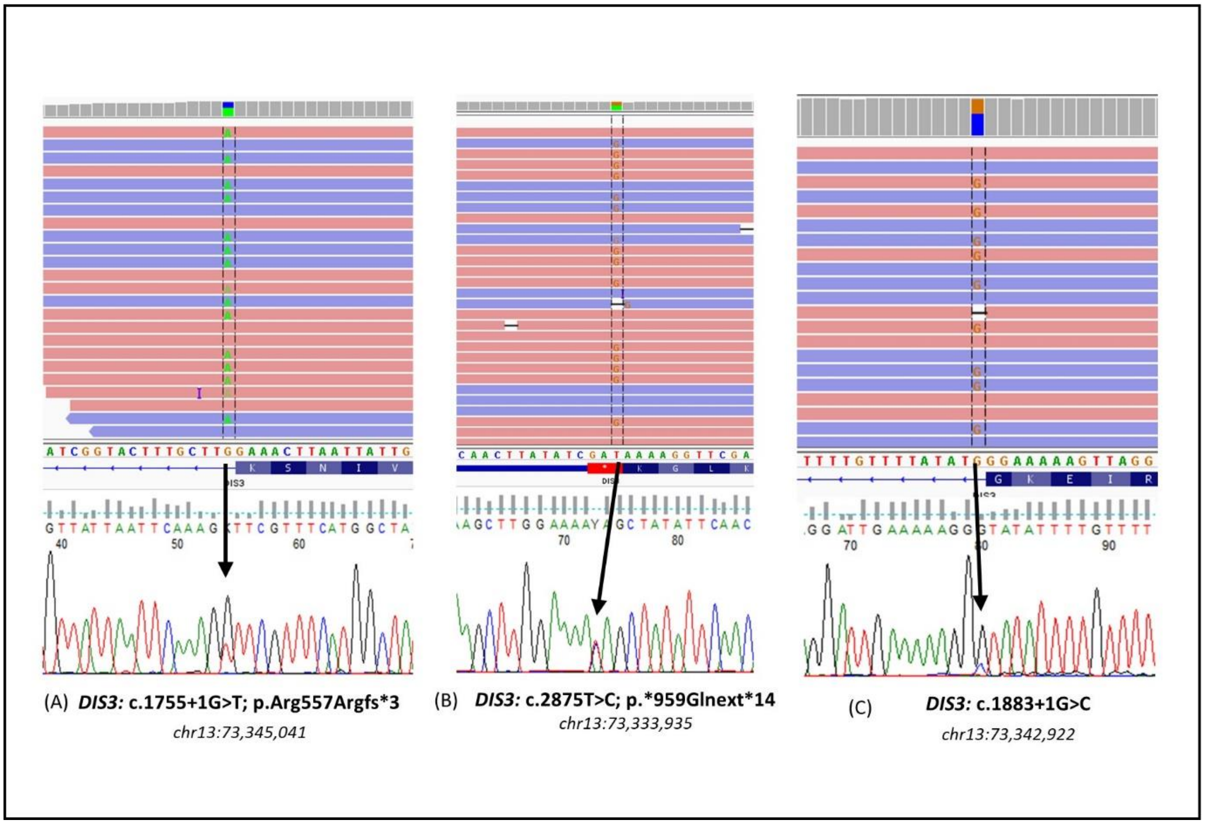

Figure S2. Quantile-quantile plots of gene-based burden tests for **(a)** rare neutral synonymous variants and **(b)** rare predicted deleterious variants across genes assessed by WES. Lambda statistics are shown on the plots. Logistic regression, with adjustment for ancestry inferred by genotype, was used to test for association between burden with individuals being defined as exposed if they carry one (or more) variants (synonymous or deleterious).

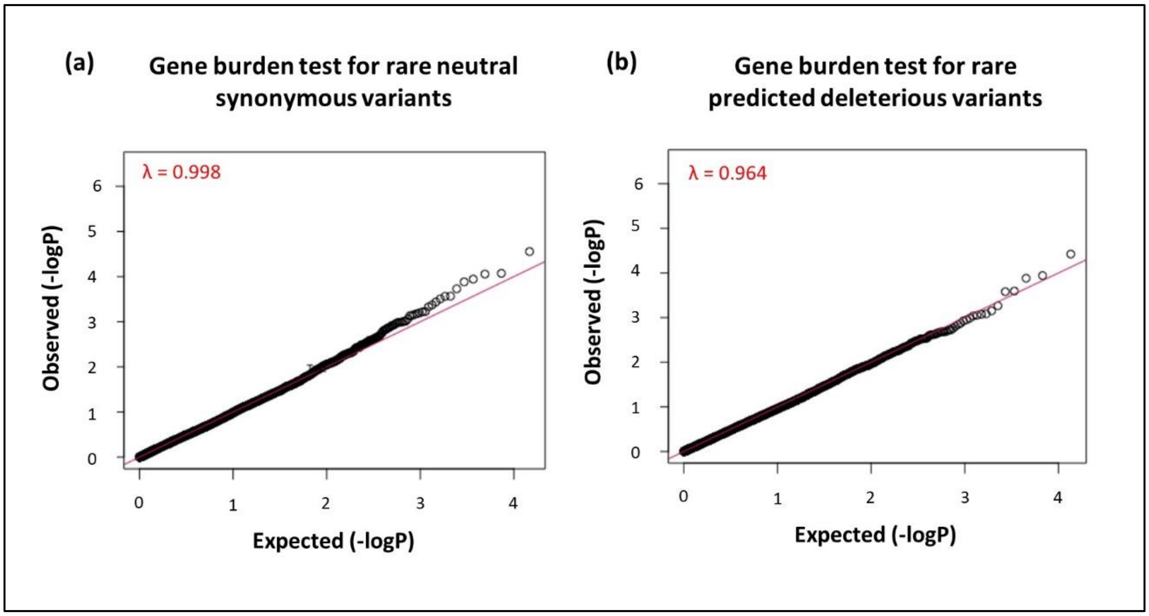

Figure S3. Mutation burden test and transcriptome analysis in carriers of *DIS3* variants. **(a)** Burden test association results for MM cases and controls carrying *DIS3* germline likely deleterious variants. OR is adjusted for ancestry inferred by genotype. **(b)** ee-MWW enrichment plots for selected significantly enriched GO and REACTOME pathways among carriers of germline likely deleterious *DIS3* variants (GO pathway Translational Termination, GO pathway ncRNA Processing, GO pathway ncRNA metabolic process and REACTOME pathway Metabolism of RNA). NES and q-values are indicated. Marked in red are genes involved in the respective pathways relative to other genes (in grey).

(a)

	Cases		Controls				
***DIS3* germline**	**Carriers**	**Non-carriers**	**Carriers**	**Non-carriers**	**OR**	**95% CI**	**P-value**
All mutations	30	751	72	3462	1.92	1.25 to 2.96	0.001
*959Gln	10[a]	751	15	3462	3.07	1.38 to 6.87	0.0007
Others	21[a]	751	57	3462	1.7	1.02 to 2.82	0.03

[a] *One patient was a compound heterozygote for *959Gln and an additional functional variant*

(b)

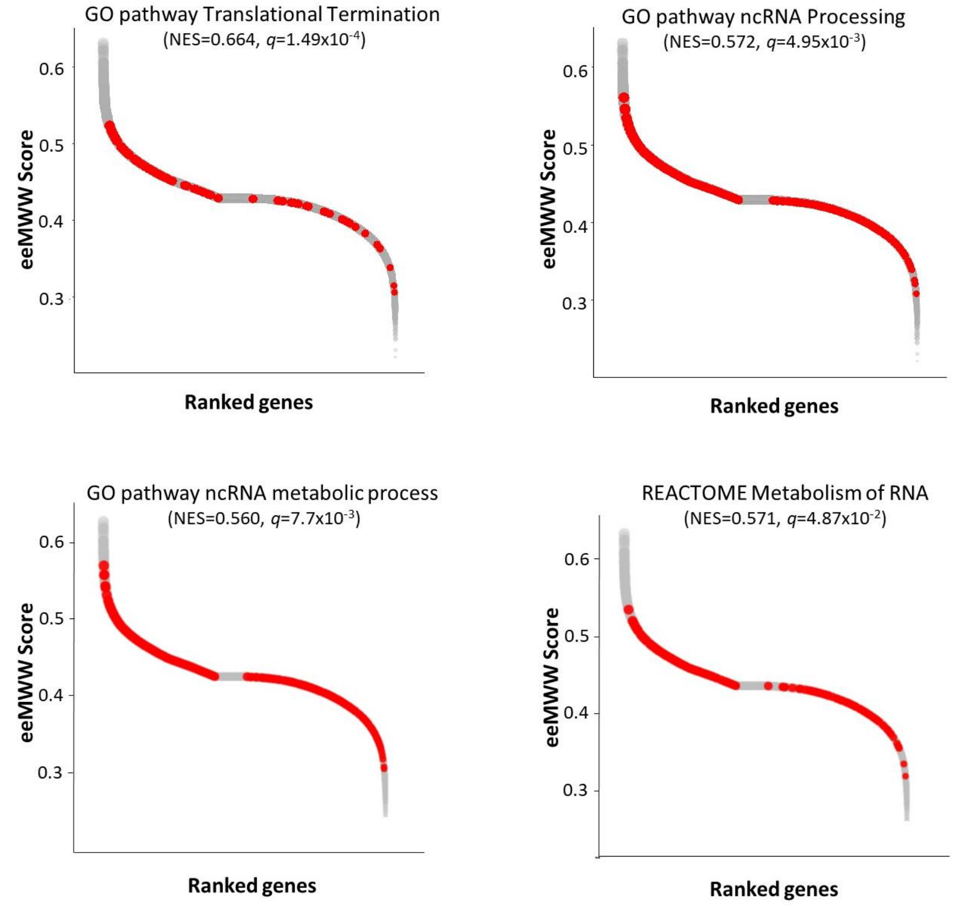

References

1 Hammerschmidt W, Sugden B. Genetic analysis of immortalizing functions of Epstein-Barr virus in human B lymphocytes. *Nature* 1989; **340**: 393–7.

2 Li H, Durbin R. Fast and accurate short read alignment with Burrows-Wheeler transform. *Bioinformatics* 2009; **25**: 1754–1760.

3 McKenna A, Hanna M, Banks E, Sivachenko A, Cibulskis K, Kernytsky A *et al.* The Genome Analysis Toolkit: a MapReduce framework for analyzing next-generation DNA sequencing data. *Genome research* 2010; **20**: 1297–303.

4 DePristo M a., Banks E, Poplin RE, Garimella K V., Maguire JR, Hartl C *et al.* A framework for variation discovery and genotyping using next- generation DNA sequencing data. *Nat Genet* 2011; **43**: 491–498.

5 Van der Auwera GA, Carneiro MO, Hartl C, Poplin R, Del Angel G, Levy-Moonshine A *et al.* From FastQ data to high confidence variant calls: the Genome Analysis Toolkit best practices pipeline. *Current protocols in bioinformatics / editoral board, Andreas D Baxevanis . [et al]* 2013; **43**: 11.10.1-33.

6 Wang K, Li M, Hakonarson H. ANNOVAR: functional annotation of genetic variants from high-throughput sequencing data. *Nucleic acids research* 2010; **38**: e164.

7 Exome variant server, NHLBI GO Exome Sequencing Project (ESP). Seattle, WA. .

8 1000 Genomes Project Consortium, Abecasis GR, Auton A, Brooks LD, DePristo MA, Durbin RM *et al.* An integrated map of genetic variation from 1,092 human genomes. *Nature* 2012; **491**: 56–65.

9 Cancer Genome Atlas Research Network, Weinstein JN, Collisson E a, Mills GB, Shaw KRM, Ozenberger BA *et al.* The Cancer Genome Atlas Pan-Cancer analysis project. *Nature genetics* 2013; **45**: 1113–20.

10 Garrison E, Marth GT. Haplotype-based variant detection from short-read sequencing. 2012; : arXiv:1207.3907 [q-GN].

11 Waller RG, Darlington TM, Wei X, Madsen MJ, Thomas A, Curtin K *et al.* Novel pedigree analysis implicates DNA repair and chromatin remodeling in multiple myeloma risk. *PLoS genetics* 2018; **14**: e1007111.

12 Wei X, Calvo-Vidal MN, Chen S, Wu G, Revuelta M V, Sun J *et al.* Germline mutations in lysine specific demethylase 1 (LSD1/KDM1A) confer susceptibility to multiple myeloma. *Cancer research* 2018. doi:10.1158/0008-5472.CAN-17-1900.

13 Frattini V, Pagnotta SM, Tala, Fan JJ, Russo M V., Lee SB *et al.* A metabolic function of FGFR3-TACC3 gene fusions in cancer. *Nature* 2018; **553**: 222–227.
